# Micromechanical Modeling of Damage Evolution and Mechanical Behaviors of CF/Al Composites under Transverse and Longitudinal Tensile Loadings

**DOI:** 10.3390/ma12193133

**Published:** 2019-09-26

**Authors:** Zhenjun Wang, Siyuan Yang, Zehui Du, Wugui Jiang, Aodi Zhang, Changchun Cai, Wei Yang

**Affiliations:** 1 National Defense Key Discipline Laboratory of Light Alloy Processing Science and Technology, Nanchang Hangkong University, Nanchang 330063, China; wangzhj@nchu.edu.cn (Z.W.); sifive@163.com (S.Y.); aodizhangnchu@163.com(A.Z.); caicc@nchu.edu.cn (C.C.); yangwei@nchu.edu.cn (W.Y.); 2 School of Materials Science and Engineering, Nanyang Technological University, Singapore 639798, Singapore; duzehui@ntu.edu.sg

**Keywords:** aluminum matrix composites, micromechanics, damage evolution, mechanical behavior.

## Abstract

This paper investigates the progressive damage and failure behavior of unidirectional graphite fiber-reinforced aluminum composites (CF/Al composites) under transverse and longitudinal tensile loadings. Micromechanical finite element analyses are carried out using different assumptions regarding fiber, matrix alloy, and interface properties. The validity of these numerical analyses is examined by comparing the predicted stress-strain curves with the experimental data measured under transverse and longitudinal tensile loadings. Assuming a perfect interface, the transverse tensile strength is overestimated by more than 180% and the transverse fracture induced by fiber failure is unrealistic based on the experimental observations. In fact, the simulation and experiment results indicate that the interface debonding arising from the matrix alloy failure dominates the transverse fracture, and the influence of matrix alloy properties on the mechanical behavior is inconspicuous. In the case of longitudinal tensile testing, however, the characteristic of interface bonding has no significant effect on the macroscopic mechanical response due to the low in-situ strength of the fibers. It is demonstrated that ultimate longitudinal fracture is mainly controlled by fiber failure mechanisms, which is confirmed by the fracture morphology of the tensile samples.

## 1. Introduction

Continuous fiber-reinforced aluminum matrix composites (CF/Al composites) have been intensively studied over the last few decades [1]. Compared with unreinforced aluminum alloys, CF/Al composites offer much higher specific mechanical strength and stiffness, higher operating temperature, and greater wear resistance [2,3]. More importantly, the properties of CF/Al composites can be tailored by tuning the microstructure parameters, including the fiber volume fraction, woven structure, and spatial/size distribution of the carbon fibers [4,5]. Liquid processing techniques, e.g., squeeze casting or gas pressure infiltration, are the most popular methods to fabricate CF/Al composites [1,6]. Aluminum melt is impregnated into fiber preform by applying high pressure until the melt solidifies. The advantages of the techniques include the uniform dispersion of carbon fibers in the matrix and the ability to produce near-net-shaped composite parts. Nevertheless, the high processing temperature can induce serious fiber-matrix interfacial reactions, which can deteriorate the mechanical properties of the CF/Al composites [7]. 

It is generally recognized that the change in the material property of the constituents in the Cf/Al composites is inevitable in most CF/Al composites fabricated by the liquid infiltration technique due to the sensitivity of the microstructure of matrix alloy to the processing conditions [8,9] and the degradation of the carbon fiber, which can be damaged by a molten aluminum alloy [10,11,12]. In particular, the chemical reaction between the fiber and matrix alloy often results in an imperfect interface with uncertain bonding strength [13,14,15]. For example, the formation of aluminum carbide (Al_4_C_3_) at the interface is beneficial for improving the bonding strength of Al/CF interfaces; however, excessive Al_4_C_3_ may impede the interface sliding, suppressing the crack propagation [7,12]. An explicit relationship between the interface characteristic and the mechanical properties of the composites has not been revealed to date. In addition, the transverse tensile properties of CF/Al composites are often very poor, although the longitudinal tensile strength and stiffness are improved with carbon fiber reinforcement [5,10]. Therefore, the use of CF/Al composites for structural applications is limited, unless the transverse direction can be reinforced by using cross-ply laminates or incorporating fabric structures [5]. However, in these CF/Al composite structures, the mechanical properties of the unidirectional CF/Al composites perpendicular to the loading direction are the weakest, which determines the overall strength and ductility of the composites’ structures [4,5,16,17,18].

In order to improve the isotropic mechanical properties and reliability of the CF/Al composites, it is important to quantitatively investigate the effect of the constituents’ characteristics on the macroscopic mechanical behavior of the composites [10] to have a better understanding of the influence of microscopic damage progress in the longitudinal and transverse fracture behavior of the composites. Complication of the interfacial behavior between the fiber and matrix is one of the challenges involved in quantifying the impact of properties’ variation on the damage and fracture response of the resulting CF/Al composites under loading. Most of the research on CF/Al composites in the literature has been focused on the interface formation mechanisms and load transfer behavior between the carbon fiber and the matrix alloy using an experimental approach. Experimental characterization of the mechanical behavior under compressive [19], tensile [10,11,16,20], bending [17,21], and fatigue loading [22] has been reported, and the dependence on the interface characteristics has been investigated in detail. Very few studies have been found that undertake a quantitative analysis of the mechanical behavior and failure mechanisms of CF/Al composites, taking into consideration the performance of the carbon fiber and Al matrix and interface. 

This work is motivated by a noticeable property degradation of the fiber and a hardening of the matrix alloy in CF/Al composites fabricated by the vacuum-assisted pressure infiltration method. The objective of this work is to investigate the progression of microscopic damage and macroscopic fracture response of CF/Al composites under transverse and longitudinal tensile conditions through a combination of experiments and numerical modeling. By assuming the constituent properties and interface bonding to be perfect or imperfect, a series of micromechanical finite element analyses have been conducted to reveal the microscopic damage evolution and the failure behavior during longitudinal and transverse tensile processes. The simulation results have been compared with the experimental stress-strain curves. From there, the contribution of matrix alloy damage, interface debonding, and fiber failure to the macroscopic fracture response is elucidated. The microscopic failure mechanism of the composites has been further discussed, with the support of the fracture morphology of the composites.

## 2. Materials and Experiment 

The CF/Al composites were fabricated with an Al-10Mg (wt. %) alloy as the matrix and unidirectional graphite fibers as the reinforcement. The chemical compositions of the Al-10Mg alloy, provided by the Beijing Institute of Aeronautical Materials Co., Ltd. (Beijing, China), are listed in Table 1. The material properties of the graphite fiber (M40J, from Toray Co. Ltd, Tokyo, Japan) are summarized in Table 2. 

**Figure 1:**
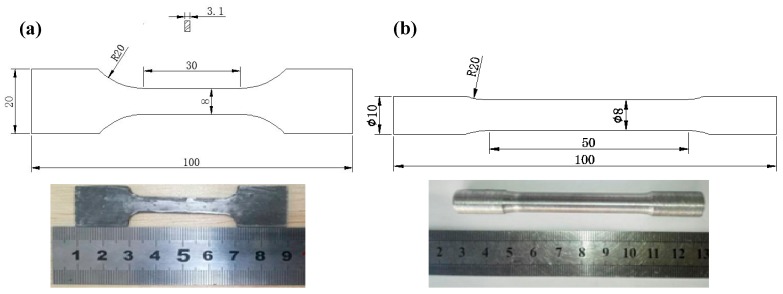
Tensile specimens of the CF/Al composite (**a**) and the as-cast Al-10 Mg alloy (**b**). The unit for the dimensions is millimeters.

The CF/Al composites were fabricated by the gas pressure infiltration (GPI) method. The detailed manufacturing process has been reported elsewhere [18,23]. The as-cast alloy, i.e., Al-10Mg alloy without fiber reinforcement, was also cast using the same processing parameters as for the CF/Al composites. The cast composite samples are thin plates with a size of 200 × 200 mm^2^. Two sets of tensile coupons (dog-bone-shaped) have been machined from the as-cast composite samples, and the dimensions of the tensile coupons can be found in Figure 1a. One set of the tensile coupons were machined along the perpendicular direction to the fiber axis for the tests with transverse tensile loading, while the other set were machined along the fiber axis for longitudinal tensile loadings. The macroscopic appearance of the composite specimens is shown in Figure 1a. The as-cast Al-10Mg ingot was cut into cylindrical tensile specimens, with the dimensions shown in Figure 1b. The samples for tensile testing are prepared with reference to Chinese Standard GB/T228-2002. Before tensile testing, the grip sections of the composite specimen were wrapped with pure aluminum plate in order to prevent early failure due to high clamping forces.

The uniaxial tensile testing of the composites and the as-cast alloy were performed on a universal mechanical testing machine (INSTRON-8801, Canton, MA, USA) at a constant loading rate of 0.5 mm/min. Microhardness tests on the as-cast alloy and the composite matrix were conducted on a STRUERS Vickers hardness tester (Copenhagen, Denmark) with a load of 245.3 mN and dwell time of 10 s, and the tests follow Chinese Standard GB/T4342-1991. The composite samples for microstructure analysis were cut along the transverse section. The samples were polished and then washed with an alcoholic solution. A Quanta200 scanning electron microscope (SEM, Hillsboro, OR, USA) was used to study the microstructural morphology of the composites. A JEM-2100F transmission electron microscope (TEM, Tokyo, Japan) was used to study the microstructure and morphology of the Cf/Al interface. NovaNanoSEM450 SEM (Hillsboro, OR, USA) was utilized to analyze the fracture surface morphology of the tensile samples after testing.

## 3. Micromechanical Model 

### 3.1. Representative Unit Cell (RUC) Model

**Figure 2:**
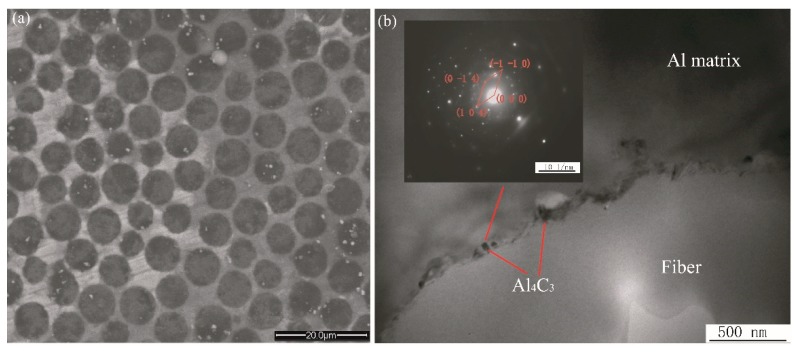
SEM micrograph of the CF/Al composite (**a**) and TEM micrograph of the fiber/matrix interface (**b**). The inset in Figure 2b is the SAED pattern taken at the Cf/Al interface.

The cross section of the CF/Al composites is illustrated in Figure 2a. There is no observable porosity or microcrack in the matrix alloy. The graphite fibers are distributed uniformly in the matrix alloy. Using Image Pro Plus software (Version 6.0, Media Cybernetics, Rockville, MD, USA), all the fibers in the SEM micrograph (Figure 2a) were identified and the fiber volume fraction (*V*_f_) in the composite can be estimated to be 56% by using the sum of fiber area divided by the total area of the micrograph in Figure 2a. Figure 2b shows a magnified TEM image of the interface between the fiber and the matrix alloy. There is a very thin interfacial layer between the fiber and matrix alloy, in which some blocky Al_4_C_3_ phase was found. Al_4_C_3_ phase was identified from the selective area electron diffraction (SAED) pattern taken at the interface (inset of Figure 2b).

**Figure 3:**
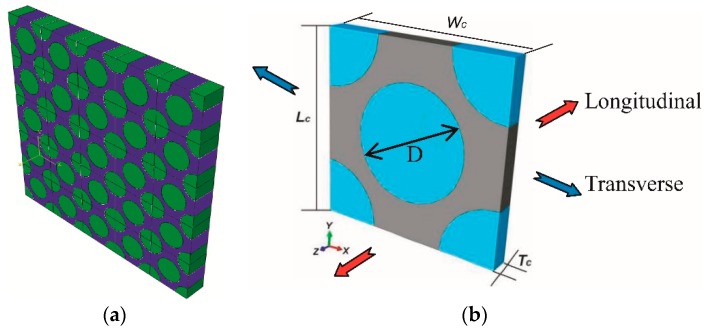
Fiber array pattern model (**a**) and RUC model (**b**).

To carry out a micromechanical simulation, an FCC (face-centered cubic) fiber array model was constructed to represent the fiber packing characteristic found in Figure 2a and schematically illustrated in Figure 3a. Figure 3b shows the RUC (representative unit cell) model, consisting of a full fiber and four quarters of a fiber. The RUC model was based on the following assumptions: (i) the fiber packing pattern is uniform and periodic; and (ii) the matrix alloy and fiber are continuum media, without a micro-void. It is a great challenge to describe the fine microstructure of the interface in the RUC model because the thickness of the interface layer is not uniform, and the orientation and morphology of the Al_4_C_3_ phase are complex (Figure 2b). Additionally, the TEM image shows that the interface thickness is extremely small compared with the fiber diameter of 6 µm and the thickness of the matrix. Therefore, the interface between the fibers and the matrix is assumed to be homogeneous, with zero thickness in the RUC model, and its mechanical behavior is mathematically described by defining the constitutive parameters such as interfacial strength and stiffness in a cohesive zone model in Section 3.2. 

In the RUC model, the length and width of the RUC (*L_C_*, *W_C_*) are 10 µm and the fiber diameter (D) is 6 µm. The fiber volume fraction of the RUC is calculated to be 56% according to the equation $V_{f}=\pi D^{2}/2L_{C}{}^{2}$. The longitudinal depth of the RUC (*T_c_*) was assumed to be 1 µm, which is not important when the periodic boundary conditions are applied to the RUC [24]. The periodic boundary conditions were applied to the RUC model as follows [25]:

(1) $$\left\{ \begin{array}{l} {\vec{u}}_{X}(0,Y,Z)-{\vec{u}}_{X}(W_{0},Y,Z)={\vec{U}}_{X}\\ {\vec{u}}_{Y}(X,0,Z)-{\vec{u}}_{Y}(X,L_{0},Z)={\vec{U}}_{Y}\\ {\vec{u}}_{Z}(X,Y,0)-{\vec{u}}_{Z}(X,Y,T_{0})={\vec{U}}_{Z} \end{array}\right.,$$

where *X*, *Y*, *Z* are the axis coordinates, and ${\vec{U}}_{i}$ (*I* = *X*, *Y*, *Z*) is the displacements applied to the nodes lying on the face in *X* = *W_C_*, *Y* = *L_C_* and *Z* = *T_C_*, respectively. ${\vec{u}}_{i}$ (*i* = *X*, *Y*, *Z*) denotes the displacement in the *X*, *Y*, *Z* direction, respectively. 

Transverse tensile loading (along the *X*-axis) is performed by applying a displacement ${\vec{U}}_{X}=(+\delta ,0,0)$ on the nodes lying on the boundary face (*X* = *W*_C_). The displacement (+*δ*) and the corresponding reaction force ($f_{i}^{X}$) of these nodes were used to calculate the transverse tensile stress-strain curves and the calculation is based on Equation (2). Longitudinal tensile loading (along the *Z*-axis) is performed by applying a displacement ${\vec{U}}_{Z}=(0,0,+\theta )$ on the nodes lying on the boundary face (*Z* = *T_C_*). The displacement (+*θ*) and reaction force ($f_{j}^{Z}$) of these nodes were used to calculate the transverse tensile stress-strain curves and their relationship is shown by Equation (3).

(2) $$\left\{ \begin{array}{l} {\bar{\varepsilon }}^{T}=\delta /W_{0}\\ {\bar{\sigma }}^{T}=\sum f_{i}^{X}/(L_{C}\cdot T_{C}) \end{array}\right.$$

(3) $$\left\{ \begin{array}{l} {\bar{\varepsilon }}^{L}=\theta /T_{C}\\ {\bar{\sigma }}^{L}=\sum f_{j}^{Z}/(L_{C}\cdot W_{C}) \end{array}\right..$$

### 3.2. Constitutive Models

#### 3.2.1. Matrix Model

**Figure 4:**
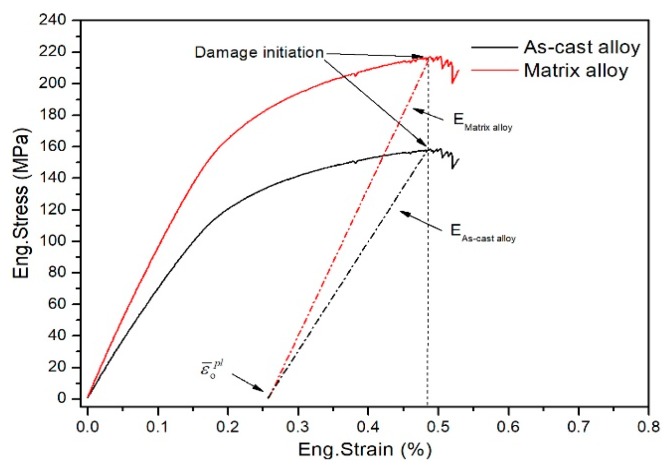
Tensile stress-strain curves of the as-cast alloy and the matrix alloy.

The mechanical properties of the matrix alloy (Al-10Mg) in the CF/Al composite should first be determined by a micromechanical modeling process. In this study, the as-cast alloy (Al-10Mg) was prepared under the same processing conditions as used for the composite. The mechanical properties of the alloy as the matrix in the composites were phenomenologically evaluated according to the difference in the microhardness of the matrix alloy and the as-cast alloy. This approach has been certified by previous research on fiber-reinforced metal matrix composites (MMCs) [26,27]. The comparison between the microhardness of the matrix alloy and the as-cast alloy is presented in Table 3. It was found that the average microhardness of matrix alloy is about 1.37 times higher than that of the as-cast alloy. The hardening of the matrix alloy can be attributed to the residual stress induced by the thermal expansion mismatch between carbon fiber and the matrix [8,28], as well as the grain refinement resulting from the high solidification rate during the gas pressure infiltration process [8]. Considering the hardening of the matrix alloy, the experimental tensile stress-strain curve of the as-cast alloy (the black line in Figure 4) was uniformly magnified by a factor of 1.37 to represent the in-situ tensile behavior of the matrix alloy, as shown in the red line in Figure 4. 

Considering the plastic characteristic of the matrix alloy, a ductile damage model was utilized to describe the damage onset and evolution behavior. It was assumed that damage was initiated when the equivalent plastic strain (${\bar{\varepsilon }}^{pl}$) reached the critical plastic strain (${\bar{\varepsilon }}_{o}^{pl}$). In the successive deformation stage, damage accumulation led to the softening of yield stress and elasticity degradation, with an increase in plastic strain. Based on the definition of equivalent plastic displacement ${\bar{u}}^{pl}$ (${\bar{u}}^{pl}=L\cdot {\bar{\varepsilon }}^{pl}$, where *L* is the characteristic length of elements), a linear softening law was adopted to formulate the damage evolution progress. With damage accumulating, the damage variable (*D*) increases from 0 to 1 monotonically, which is defined by the following formula [29]:

(4) $$D=\frac{{\bar{u}}^{pl}}{L\cdot {\bar{\varepsilon }}_{f}^{pl}},$$

where ${\bar{\varepsilon }}_{f}^{pl}$ is the equivalent plastic strain corresponding to failure initiation. Based on the tensile stress-strain curves (Figure 4), the elastic constants and ductile damage parameters were determined and listed in Table 4. The critical plastic strain for damage initiation (${\bar{\varepsilon }}_{o}^{pl}$) is determined to be 0.25% (Figure 4). The critical plastic strain for failure initiation (${\bar{\varepsilon }}_{f}^{pl}$) is usually assumed to be three times the ${\bar{\varepsilon }}_{o}^{pl}$ [24], resulting ${\bar{\varepsilon }}_{f}^{pl}=0.75\%$. The plastic flow behaviors of the matrix alloy and as-cast alloy were defined by the stress-strain data measured from the tensile stress-strain curves in Figure 4.

#### 3.2.2. Fiber Model

The graphite fiber can be assumed to be linearly elastic with transversely isotropic material behavior. Before failure, the longitudinal and transverse elastic properties of the fiber are characterized by Young’s modulus $E_{L}$ and $E_{T}$, Poisson’s ratios $\nu_{LT}$ and $\nu_{TT}$, shear modulus $G_{LT}$ and $G_{TT}$, respectively; these parameters are listed in Table 5 [30,31]. The transversely isotropic strength of the fiber is defined by the mechanical parameters, including the tensile strength $S_{L}^{t}$, compression strength $S_{L}^{c}$, shear strength $\tau_{LT}$ in longitudinal direction, and the tensile strength $S_{T}^{t}$, compression strength $S_{T}^{c}$, shear strength $\tau_{TT}$ in transverse direction. In a previous work [12], it was found that the average strength of the carbon fibers in composites is 1760 MPa, which is about 0.4 times the strength of the original fiber (4400 MPa). Considering the significant degradation in the fiber strength, all the in-situ fiber strength (for the fibers in the composites) is assumed to be 0.4 times the original fiber strength parameters, as shown in Table 6.

As the transversely isotropic characteristics of the carbon fiber are similar to those of the unidirectional reinforced composite laminate, Tsai-Wu failure criterion, which has been used to determine the mechanical failure of fiber-reinforced composite laminate [32], was modified to define the fiber failure behaviors in CF/Al composites and can be described as in Equation (5):

(5) $$\begin{array}{l} F_{1}(\sigma_{1}+\sigma_{2})+F_{3}\sigma_{3}+F_{11}(\sigma_{1}{}^{2}+\sigma_{2}{}^{2})+F_{33}\sigma_{3}{}^{2}+2F_{12}\sigma_{1}\sigma_{2}+2F_{23}(\sigma_{1}+\sigma_{2})\sigma_{3}\\ +F_{44}(\tau_{23}{}^{2}+\tau_{13}{}^{2})+F_{66}\tau_{12}{}^{2}>1 \end{array}$$

where, $F_{1}=1/S_{T}^{t}-1/S_{T}^{c}$, $F_{3}=1/S_{L}^{t}-1/S_{L}^{c}$, $F_{11}=1/S_{T}^{t}S_{T}^{c}$, $F_{33}=1/S_{L}^{t}S_{L}^{c}$, $F_{12}=-1/2S_{T}^{t}S_{T}^{c}$, $F_{23}=-1/2{\left(S_{T}^{t}S_{T}^{c}S_{L}^{t}S_{L}^{c}\right)}^{1/2}$, $F_{44}=1/\tau_{LT}^{2}$, $F_{66}=1/\tau_{TT}^{2}$. These strength coefficients were calculated according to the strength parameters in Table 6. By programming a user-defined material subroutine in FORTRAN code, the fiber failure criterion (Equation (5)) can be implemented and interfaced with the ABAQUS standard.

When the stress state at the integration point satisfies Equation (5), the stiffness at each failure integration point is degraded. The degradation was applied to the elastic modulus in Table 5 by multiplying them by a positive discount factor less than 1.0. The elastic behavior of the failed fiber is governed by the modified linear elastic law for transversely isotropic material in the following:

(6) $$\left[\begin{array}{c} \varepsilon_{1}\\ \varepsilon_{2}\\ \varepsilon_{3}\\ \gamma_{23}\\ \gamma_{31}\\ \gamma_{12} \end{array}\right]=\left[\begin{array}{cccccc} 1/\lambda E_{T} & -\nu_{TT}/\lambda E_{T} & -\nu_{LT}/\lambda E_{L} & 0 & 0 & 0\\ -\nu_{TT}/\lambda E_{T} & 1/\lambda E_{T} & -\nu_{LT}/\lambda E_{L} & 0 & 0 & 0\\ -\nu_{LT}/\lambda E_{T} & -\nu_{LT}/\lambda E_{T} & 1/\lambda E_{L} & 0 & 0 & 0\\ 0 & 0 & 0 & 1/\xi G_{LT} & 0 & 0\\ 0 & 0 & 0 & 0 & 1/\xi G_{LT} & 0\\ 0 & 0 & 0 & 0 & 0 & 1/\xi G_{TT} \end{array}\right]\left[\begin{array}{c} \sigma_{1}\\ \sigma_{1}\\ \sigma_{1}\\ \tau_{23}\\ \tau_{31}\\ \tau_{12} \end{array}\right],$$

where λ and ξ are the degradation factor of the elastic and shear modulus, respectively. Previous studies have shown that the assumption of λ=0.01 and ξ=0.2 can account for the property degradation of the failed fibers [33].

#### 3.2.3. Interface Model

**Figure 5:**
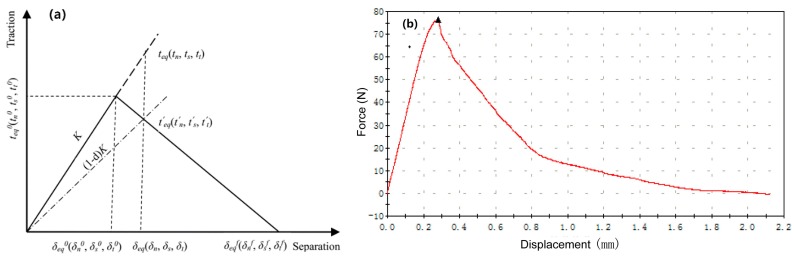
Schematic illustration of the traction-separation law for the cohesive zone model (**a**) and force-displacement curve of the fiber pullout test (**b**).

It is widely accepted that interface failure is the most typical failure mechanism in unidirectionally reinforced composites, especially under transverse tensile loading. The impact of the interface is generally considered through the assumption that the interface bonding is perfect or imperfect. In this study, a cohesive zone model was utilized to describe the debonding behavior of the imperfect interface. The behavior of cohesive zone elements was usually described by a bilinear traction-separation law [29]. It is assumed that the stress increases linearly with the increase of displacement before the interface strength is reached, as shown in Figure 5a. The interfacial stiffness *K* is assumed to be proportional to the interface material elastic modulus $E_{I}$, and to be inversely proportional to the interface thickness $\delta_{I}$. *E*_I_ is assumed as the average of the elasticity modulus of the matrix alloy and the fiber, i.e., $E_{I}=\left(E_{m}+E_{f}^{T}\right)/2$. $\delta_{I}$ is defined as $\delta_{I}=kd_{f}$, where *k* is assumed as the ratio of interface thickness to the fiber diameter ($d_{f}$). Therefore, the interfacial stiffness can be defined as $K=\left(E_{m}+E_{f}^{T}\right)/2kd_{f}$. According to [24] and [34], both the transverse and the longitudinal tensile behavior of the composites can be well simulated by setting *k* at 0.05, which represents a very thin interface between the matrix and fibers [12].

A maximum nominal stress criterion was adopted to determine the initiation of interface damage, which can be expressed as follows:

(7) $$Max\{\frac{\langle t_{n}\rangle }{t_{n}{}^{0}},\frac{t_{s}}{t_{s}{}^{0}},\frac{t_{t}}{t_{t}{}^{0}}\}=1,$$

where $t_{n},t_{s},t_{t}$ are the normal and two shear stress components at the interface. $t_{n}{}^{0},t_{s}{}^{0},t_{t}{}^{0}$ are the critical nominal stress values corresponding to the initiation of the damage at interface. The Macaulay bracket 〈〉 implies that purely compressive stress does not induce the damage initiation. The interfacial shear strength was measured by a fiber pullout testing method [35,36]. The graphite fiber bundle with a uniform length of 2.5 mm and embedded in the Al-10Mg alloy was pulled out using an Instron 5540 testing machine, and the resultant force-displacement curve was presented in Figure 5b. By measuring the number of pullout fibers (*n*) and their average length (*L*), the interfacial shear strength was calculated by $t_{s}^{0}=t_{t}^{0}=F_{\max}/n\cdot \pi d_{f}L$, which results in an interfacial shear strength of 9.5 MPa. For the as-studied CF/Al composites, the interface bonding strength is very low. As the transverse tensile property is directly related to the interfacial bonding strength [10,22], the interfacial bonding strength $t_{n}^{0}$ = 16 MPa was adopted in the simulations according to the transverse tensile tests conducted in [10].

When the interfacial damage is activated, a scalar damage evolution variable (d) is introduced to define the degradation rate of the interfacial stiffness (K). The damage evolution variable (d), which increases from 0 to 1 monotonically, is defined by a damage evolution model based on the dissipated energy $G_{c}=0.5\delta_{eq}^{f}\cdot t_{eq}^{0}$, where $\delta_{eq}^{f}$ is the equivalent displacement at interfacial failure, and $t_{eq}^{0}$ is the equivalent stress for the initiation of interfacial damage. In [26], the cohesive zone parameter of $G_{c}$, for the unidirectional fiber-reinforced aluminum matrix composites with a transverse strength of 189.9 MPa, has been calibrated from the simulation, resulting in $G_{c}=75\;J/m^{2}$. In this study, the transverse strength of the CF/Al composite is about 25.2 MPa. Hence, the value of $G_{c}$ for the CF/Al composite is set to 7.5 $J/m^{2}$. Given the equivalent stress at interfacial damage initiation $t_{eq}^{0}=\sqrt{{\left(t_{s}^{0}\right)}^{2}+{\left(t_{t}^{0}\right)}^{2}+{\left(t_{n}^{0}\right)}^{2}}=20.9\;\mathrm{MPa}$, the equivalent displacement at interfacial failure $\delta_{eq}^{f}$ can be determined to be 0.72 × 10^-6^ m. According to the work on metal matrix composite (MMCs) done by Needleman et al. [37], the equivalent displacement $\delta_{eq}^{f}$ is typically assumed to be 7-9 times that of the critical displacement $\delta_{eq}^{0}$; as a result, the equivalent displacement at interfacial damage initiation $\delta_{eq}^{0}$ was set as 0.08 × 10^−6^ m in the cohesive zone model.

## 4. Results and Discussion

In this work, four micromechanical finite element analysis (MFEA) models were utilized to analyze the transverse and longitudinal tensile behavior. The settings of the constituents and interface in these MFEA models are listed in Table 7. The material parameters of the matrix alloy in the MFEA-A, MFEA-B, and MFEA-D models are set as the data of the matrix alloy listed in Table 4, whereas the matrix material parameters in the MFEA-C model are set to those of the as-cast alloy (Table 4). The imperfect interface in the MFEA-A, MFEA-C, and MFEA-D models was described by the cohesive zone model, which is defined by setting the constitutive parameters in the bilinear traction-separation law to those described in Section 3.2.3, while the perfect interface in the MFEA-B model is set by tying the fiber surface to the matrix surface in ABAQUS. The fiber properties in the MFEA-A, MFEA-B, and MFEA-C models are assumed to be those of the in situ fiber (Table 6), whereas the fiber properties in the MFEA-D model are assumed to be those of the original fiber (Table 6). 

### 4.1. Transverse Tensile Behavior

**Figure 6:**
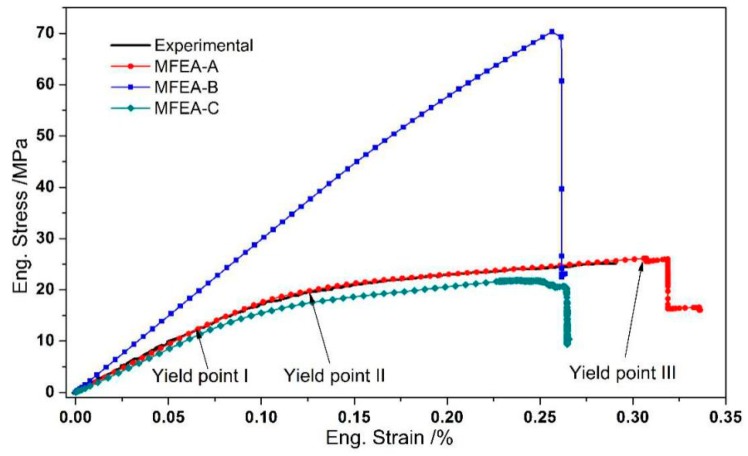
Experimental and calculated tensile stress-strain curves of the CF/Al composites.

Under the transverse tensile loading condition, the deformation behavior of the composites was tested, and the macroscopic mechanical response was simulated using the four MFEA models. Figure 6 presents the experimental tensile stress-strain curve and the stress-strain curves calculated with the MFEA-A, MFEA-B, and MFEA-C models. It was found that the stress-strain curve calculated by the MFEA-A model almost overlaps with the experimental curve in the entire transverse tensile process. The stress-strain curve calculated by the MFEA-C model also follows the experimental data closely. However, the stress-strain curve calculated by the MFEA-B model differs significantly from the experimental curve. Therefore, the MFEA-A model more accurately describes the transverse tensile behavior of the composites. 

**Figure 7:**
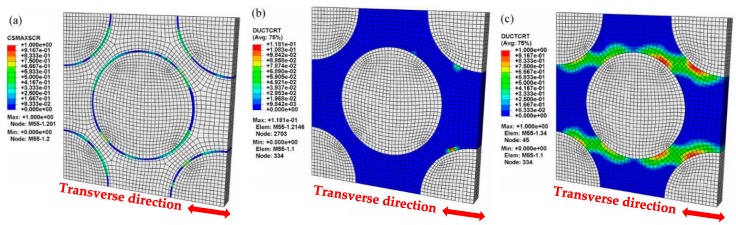
Microscopic damage and failure in the transverse tensile loading process in the MFEM-A model: (**a**) interface failure ($\varepsilon_{T}$ = 0.07%); (**b**) matrix damage initiation ($\varepsilon_{T}$ = 0.12%); (**c**) matrix failure ($\varepsilon_{T}$ = 0.31%).

**Figure 8:**
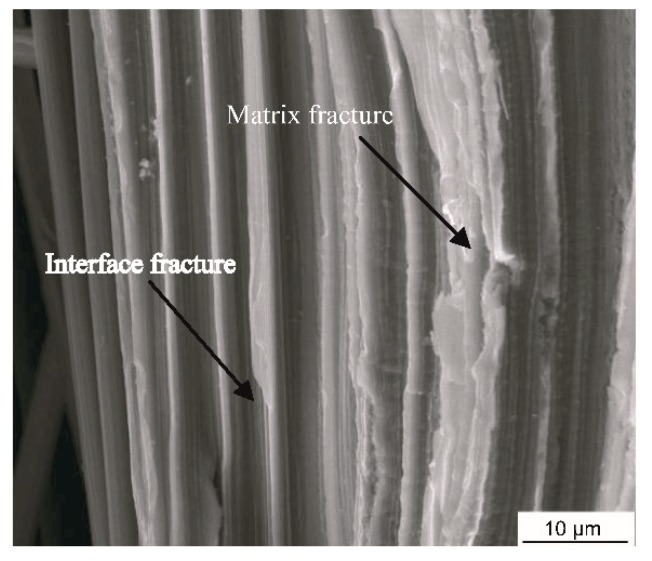
Fracture morphology of the composites subjected to transverse tensile loading.

Figure 7 presents the microscopic damage and failure behavior of the matrix and interface during the transverse tensile process simulated by the MFEM-A model. The tensile strain at the initiation of interface failure, matrix damage and matrix failure are 0.07%, 0.12%, and 0.31%, which are denoted by the yield points I, II, and III in the stress-strain curve calculated by the MFEM-A model (Figure 6), respectively. At the transverse strain ($\varepsilon_{T}$) of 0.07%, the tangent modulus of the calculated stress-strain curve is slightly reduced (yield point I in Figure 6). By examining the numerical simulation results, we found that yield point I corresponds to the initiation of local interface failure, which is illustrated in Figure 7a. After yield point I, the engineering stress increases monotonously with the increase in transverse strain, but the tangent modulus decreases continuously. At a transverse strain of 0.12%, there is a significant decline in the tangent modulus (yield point II in Figure 6). This can be attributed to the local damage initiation in the matrix alloy adjacent to the interface (Figure 7b). Hereafter, the increase in plastic strain leads to damage accumulation in the matrix alloy and the engineering stress increases slowly, as displayed by the segment between yield points II and III in Figure 6. When the applied transverse strain reaches 0.31%, the ductile damage variable in Figure 7c becomes 1.0 in some elements, which indicates that element failure has taken place in the local matrix alloy near the interface. This corresponds to yield point III in Figure 6. After that, with the strain increasing, the stress increases slightly and then drops dramatically, which implies the eventual fracture of the composite. It can be concluded that the interface debonding, which was induced by the matrix alloy failure in the vicinity of the interface, is the main failure mechanism for the CF/Al composites under transverse tensile loading condition. This is supported by the fracture surface of the CF/Al composites obtained in the transverse tensile experiment (shown in Figure 8). 

**Figure 9:**
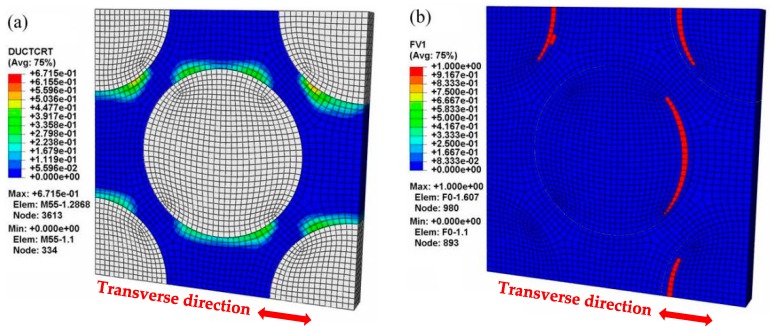
Microscopic damage and failure at the final stage of the transverse tensile process (MFEM-B model): (**a**) matrix damage ($\varepsilon_{T}$ = 0.26%); (**b**) fiber fracture ($\varepsilon_{T}$ = 0.26%).

The macroscopic mechanical behavior calculated by the MFEA-B model was found to deviate from the experimental stress-strain curve (Figure 6). In this case, the relationship between the stress and strain appears nearly linear elastic over the whole transverse strain range. In the MFEA-B model, the interface bonding was assumed to be perfect. Without interface debonding in transverse tensile loading, the carbon fibers can bear most of the transverse load when the interface remains intact. The transverse tensile strength calculated based on this model represents an upper bound, which is overestimated by more than 180%. This has previously been observed in numerical simulations of alumina fiber-reinforced aluminum composites, where the simulation based on the assumption of perfect interface does not correctly depict the failure behavior in transverse tensile loading [26]. In the current study, however, it is noted that the macroscopic stress-strain curve (MFEA-B in Figure 6) shows a suddenly drop at a strain of 0.26%, which implies transverse tensile failure. In fact, the ultimate failure can be attributed to fiber fracture rather than matrix alloy failure, as displayed in Figure 9. This is also confirmed by the fact that the calculated transverse strength (around 70 MPa in Figure 6) is similar to the in situ transverse tensile strength of the fiber as listed in Table 6. Comparing the simulation results of MFEM-A and MFEM-B, it is further demonstrated that the interface debonding induced by matrix failure is responsible for the significant reduction of the overall strength in the transverse tensile process. 

Comparing the stress-strain curve calculated by the MFEM-C model with the experimental measurement, one observes that the calculated curve is below the experimental curves during the entire test process, as shown in Figure 6. This is due to the fact that the mechanical properties of the matrix alloy in the MFEM-C model are assumed to be those of the as-cast alloy rather than of the in situ alloy. Compared to the influence of interface bonding on the mechanical behavior, however, it was found that the mechanical properties of the matrix alloy hardly alter the characteristics of the stress-strain curve under the transverse tensile condition.

### 4.2. Longitudinal Tensile Behavior

**Figure 10:**
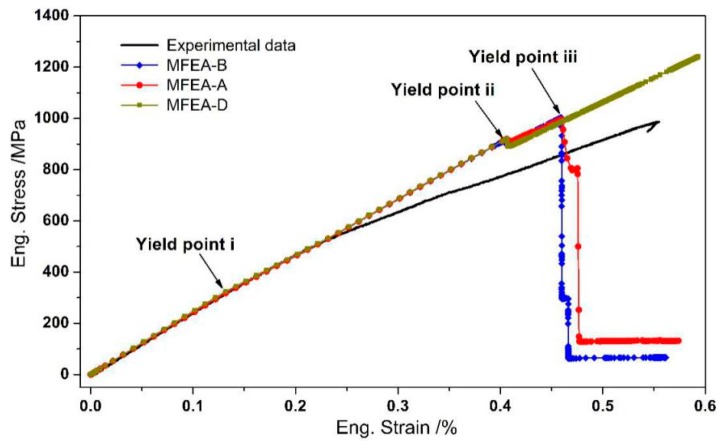
Experimental and calculated stress-strain curves in the longitudinal tensile process.

Figure 10 shows the experimental stress-strain curve along with the macroscopic response curves simulated by the different MFEA models under longitudinal tensile loading conditions. It is noticed that all the calculated curves are in line with the experimental curve in the initial deformation stage, where the tensile strain ranges from zero to 0.23%. When the longitudinal tensile strain exceeds 0.23%, however, all the calculated stress-strain curves begin to deviate from the experimental curve. At the ultimate tensile deformation stage, the fracture strength predicted by the MFEA-A model is equal to the value predicted by the MFEA-B model, both of which are relatively closer to the experimental value, compared to that predicted by the MFEA-D model. In the MFEA-D model, no failure is observed, even when the strain is over 0.6%. This is somewhat unreasonable. Therefore, further analysis of the composite failure mechanism will be based on the MFEA-A model in the following. 

**Figure 11:**
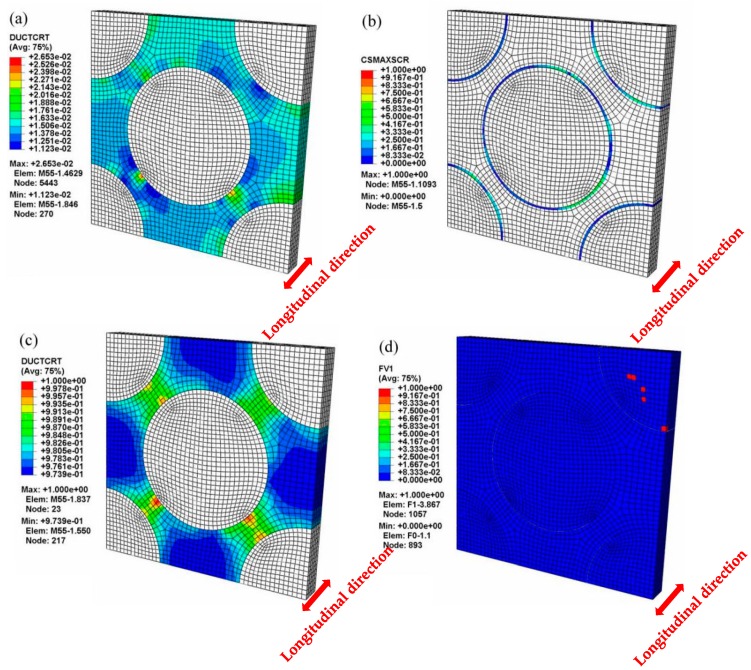
Microscopic damage and failure in the longitudinal tensile process (MFEA-A). (**a**) Matrix damage initiation ($\varepsilon_{L}$ = 0.13%); (**b**) local interface failure ($\varepsilon_{L}$ = 0.39%); (**c**) local matrix failure ($\varepsilon_{L}$ = 0.40%); (**d**) local fiber facture ($\varepsilon_{L}$ = 0.46%).

**Figure 12:**
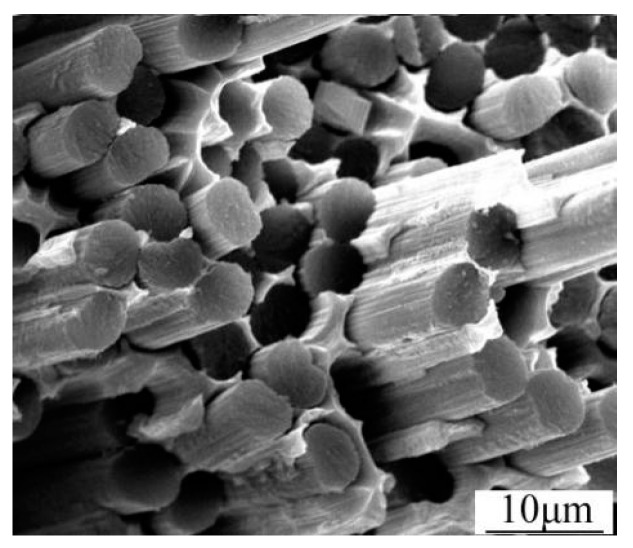
Fracture morphology of the composites subjected to longitudinal tensile loading.

Under the longitudinal tensile loading, the progressive damage and fracture behaviors of the constituents in the composites simulated by the MFEA-A model are presented in Figure 11. The longitudinal tensile strains ($\varepsilon_{L}$), indicative of the initiations of the matrix damage, matrix failure, and fiber fracture, are ~0.13%, ~0.40%, and ~0.46%, respectively, which are marked as yield points i, ii, and iii in the curve calculated by the MFEA-A model (Figure 10). These failure strains are determined by correlating the strains with the fracture/damage events in the simulation. At a longitudinal strain ($\varepsilon_{L}$) of 0.13%, it has been found that the damage initiation point occurred in the matrix alloy near the interface (Figure 11a). The damage onset in the matrix alloy leads to a slight decrease in the tangent modulus of the stress-strain curve and results in the first yield point (yield point i in Figure 10). With the increase in tensile strain, the plastic damage accumulates in the interface zone, triggering a fiber/matrix separation process, which leads to local interface failure (Figure 11b). Almost simultaneously, it is found that local matrix failure begins to appear in the vicinity of the interface (Figure 11c). The local failure of the interface and matrix alloy contributes to a significant stress fluctuation (at a strain of 0.4%) on the stress-strain curve (yield point ii in Figure 10). As the longitudinal loading continues to increase, the ductile damage in matrix alloy develops continuously, which results in more and more interface failure due to the insufficient interfacial bonding strength. Concurrently, the equivalent stress on the fibers increases quickly during this period. Finally, the failure point occurs in the fiber element that is adjacent to the interface (Figure 11d). The local fiber fracture induces a sudden stress reduction in the stress-strain curve (yield point iii in Figure 10), and eventually results in overall macroscopic failure. As such, it can be concluded that the fibers are able to bear most of the longitudinal load and the ultimate fracture of the composites is dominated by fiber failure mechanism. Therefore, the fiber pullout induced by interface failure ought to be observable in the fracture surface under longitudinal tensile loading conditions. This is supported by the fracture morphology of the composites, captured by the SEM micrograph shown in Figure 12.

It should be noted that the stress-strain curve calculated by the MFEA-D model (in Figure 10) does not display any stress reduction, even though the tensile strain exceeds 0.6%. This implies that overall fracture would not take place in the simulation, assuming the fiber property is same as that of original carbon fibers. The ultimate fracture strength calculated by the MFEA-D model is far greater than the experimental results and the results simulated by the MFEA-A and MFEA-B models. This implies that the fiber failure mechanism dominates the composite failure under the longitudinal tensile loading conditions, not CF/Al interface bonding (perfect or imperfect). 

### 4.3. Simulation Error Analysis

Comparing the curves calculated by the different models with the experimental data ( Figure 6; Figure 10), it is suggested that the MFEA-A model can reproduce the tensile deformation behaviors of the composites more accurately than the other three models, regardless of transverse or longitudinal tensile loading conditions. It is necessary to evaluate the calculation error of this model and give an explanation for the deviation between the experimental and simulating curves. Table 8; Table 9 summarize the experimental and calculated mechanical properties of the composites under transverse and longitudinal tensile conditions, respectively. The experimental data were obtained from six composite samples (three from transverse tests and three from longitudinal testes) of the same size (Figure 1a). Under both transverse and longitudinal tensile conditions, the relative errors of the ultimate tensile strength (UTS*_T_*, UTS*_L_*) and the elastic modulus ($E_{T}^{C}$ (at strain of 0.07%), $E_{L}^{C}$ (at strain of 0.13%)), are very small (less than 5%). However, the calculation errors of the fracture strain are relatively high, particularly the calculation error of the longitudinal fracture strain, which is as high as 16.4% (Table 9). Moreover, the calculated stress-strain curve for longitudinal loading did not follow the experimental data when the tensile strain was over 0.23% (Figure 10).

The large calculation errors can originate from the vacuum-assisted pressure infiltration processes used to fabricate the CF/Al composite. During the manufacturing process, it is inevitable that some fibers are not perfectly aligned along the orientation of the unidirectional composite [12]. When the composites are subjected to longitudinal tensile loading, these misaligned fibers realign themselves slightly to the longitudinal loading direction. These microscopic deflections and adjustments of fibers result in a macroscopic stress softening phenomenon, as shown in the experimental stress-strain curve under the longitudinal tensile condition (Figure 10). In the MFEA models, however, we assumed that the fibers are arranged in line with the longitudinal tensile direction. The deflection and adjustment of fibers could not be reflected in the simulation. As a result, no stress-softening behavior is observed in the calculated curve, and a lower fracture strain is obtained as compared with that in the experimental curve. This could be the reason for the deviation of the calculated stress-strain curve from the experimental curve when the longitudinal tensile strain is over 0.23% (Figure 10). 

It is also noteworthy that the actual microstructure in the composites is not fully dense. In our experience, there are always some micropores presented in the matrix alloy of the composites fabricated by the vacuum-assisted pressure infiltration method [18,38]. These microstructural defects result in a degradation in the ductility of the matrix alloy, exerting great influence on the transverse tensile deformation behavior of the composite. Therefore, the fracture strain of the composite measured under transverse tensile loading is lower by δ*_T_* = +5.2% than that predicted by the MFEA-A model, in which the effect of microscopic porosities is not considered. 

## 5. Conclusions

The microscopic damage evolution and macroscopic fracture behavior of the CF/Al composites under transverse and longitudinal tensile loading have been investigated using micromechanical finite element analysis and experimental methods. The simulation results indicate that the damage progression and the macroscopic mechanical response are strongly influenced by the material mechanical properties of the constituents (fiber, matrix alloy) and the interface bonding. Essentially, the mechanical property of the matrix alloy does not have a significant influence on the transverse tensile behavior and transverse strength, while the interface debonding induced by the matrix damage accumulation plays an important role in the course of transverse tensile fracture. During the longitudinal tensile process, however, the ultimate fracture of the composite is dominated by the fiber failure mechanism rather than by interface debonding. Even in the case of perfect interface bonding (the MFEA-B model), the simulation result shows low fracture strength due to the insufficient in situ tensile strength of the fiber, and vice versa—the tensile strength is much higher if the original fiber strength is used in simulation. This indicates that the longitudinal tensile strength is determined by the strength of the in situ fiber, and it is vital to prevent the mechanical property degradation of graphite fiber during the composites’ preparation.

## Figures and Tables

**Table 1:** Chemical composition of the Al-10Mg alloy (wt. %).

Element	Mg	Si	Cu	Mn	Ti	Al
Content	9.5-11.0	0.3	0.1	0.15	0.15	Balance

**Table 2:** Primary properties of the graphite fiber M40J.

Fiber Type	Elastic Modulus/GPa	Poisson Ratio	Tensile Strength/MPa	Density/g·cm^−3^	Elongation/%	Fiber Diameter/μm	Fiber Length/mm
M40J	377	0.26	4400	1.77	0.7	6	200

**Table 3:** Microhardness of the as-cast alloy and the matrix alloy.

Materials	Microhardness					
	Test 1	Test 2	Test 3	Test 4	Test 5	Average
As-cast alloy	106.0 ± 2	92.2 ± 1	116.0 ± 3	96.4 ± 1	105.0 ± 3	103.1 ± 2
Matrix alloy	146.0 ± 1	143.0 ± 1	135.0 ± 2	134.0 ± 2	152.0 ± 3	142.0 ± 1.8

**Table 4:** Mechanical properties of the as-cast alloy and the matrix alloy.

	E/GPa	ν	$\sigma_{s}/\mathrm{MPa}$	${\bar{\varepsilon }}_{o}^{pl}/\%$	${\bar{\varepsilon }}_{f}^{pl}/\%$
As-cast alloy	66.31	0.33	100.35	0.25	0.75
Matrix alloy	90.84	0.33	137.48	0.25	0.75

**Table 5:** Elastic constants of the graphite fiber.

Elastic Parameters	$E_{L}/\mathrm{GPa}$	$E_{T}/\mathrm{GPa}$	$\nu_{LT}$	$\nu_{TT}$	$G_{LT}/\mathrm{GPa}$	$G_{TT}/\mathrm{GPa}$
Properties	377	19	0.26	0.3	8.9	7.3

**Table 6:** Strength parameters of the original and in-situ fibers.

Strength Parameters	$S_{L}^{t}/\mathrm{MPa}$	$S_{L}^{c}/\mathrm{MPa}$	$S_{T}^{t}/\mathrm{MPa}$	$S_{T}^{c}/\mathrm{MPa}$	$\tau_{LT}/\mathrm{MPa}$	$\tau_{TT}/\mathrm{MPa}$
Original fiber	4400	2250	175	590	340	239
In-situ fiber	1760	900	70	236	136	96

**Table 7:** Setting of the matrix, fiber and interface in the MFEA models.

Models	Matrix Properties Set as	Cf/Al Interface	Fiber Properties Set as
MFEA-A	Matrix alloy	Imperfect	In-situ fiber
MFEA-B	Matrix alloy	Perfect	In-situ fiber
MFEA-C	As-cast alloy	Imperfect	In-situ fiber
MFEA-D	Matrix alloy	Imperfect	Original fiber

**Table 8:** Experimental and calculated mechanical properties under transverse tensile loading.

Mechanical Property	UTS*_T_*/MPa	$E_{L}^{C}/\mathrm{GPa}$	δ*_T_*/%
Experimental	25.17 ± 0.55	17.98 ± 0.41	0.291 ± 0.020
Calculated (MFEA-A)	26.15	18.11	0.306
Relative error	+3.9%	+0.7%	+5.2%

**Table 9:** Experimental and calculated mechanical properties at longitudinal tensile loading.

Mechanical Property	UTS*_L_*/MPa	$E_{L}^{C}/\mathrm{GPa}$	δ*_L_*/%
Experimental	985.8 ± 7.7	199.7 ± 3.2	0.55 ± 0.11
Calculated (MFEM-A)	995.8	208.2	0.46
Relative error	+1.0%	+4.3%	−16.4%
